# Strangulation: A Cause or Mimicker of Global Myocardial Hypoxia on ECG

**DOI:** 10.7759/cureus.25139

**Published:** 2022-05-19

**Authors:** Damir Vukomanovic, Abdulbaril Olagunju, Farouk Mookadam, Michael Zawaneh, Samuel Unzek

**Affiliations:** 1 Internal Medicine, University of Arizona College of Medicine, Phoenix, USA; 2 Internal Medicine, Creighton University School of Medicine, Phoenix, USA; 3 Cardiology, University of Arizona College of Medicine, Phoenix, USA; 4 Cardiology/Electrophysiology, University of Arizona College of Medicine, Phoenix, USA

**Keywords:** st-segment resolution, st-segment changes, mimicking st elevation, avr lead, electrocardiogram (ecg/ekg)

## Abstract

ST segment changes are often associated with myocardial ischemia but may be mimickers. We present a 21-year-old male who suffered a cardiac arrest following a suicide attempt by strangulation. Initial ECG revealed diffuse ST depressions and ST elevation in augmented vector right (aVR), concerning myocardial ischemia. However, repeat ECG revealed normal ST segments and an echocardiogram revealed no wall motion abnormalities. This case highlights the effects of systemic hypoxia on cardiac muscle and the need for a broad differential diagnosis when interpreting an ECG. This is invaluable when ST segment changes mimic acute myocardial infarction, but the clinical scenario suggests global hypoxia.

## Introduction

Global myocardial hypoxia can be classified as ischemic, systemic, anemic, and histotoxic hypoxia [1-6]. Ischemic hypoxia results from compromised myocardial blood supply [3] as in non-ST elevation acute coronary syndrome, posterior ST elevation myocardial infarction (STEMI), STEMI with reciprocal ST-depression (STD), Wellen’s syndrome, or de Winter’s pattern [4-7]. Systemic hypoxia is due to a drop in arterial oxygen tension (PaO_2_) in the presence of adequate perfusion such as any form of respiration compromise including strangulation [3]. Anemic and histotoxic hypoxia are less common and represent an impaired blood oxygen transport mechanism in the presence of normal PaO_2_ and decreased tissue utilization of oxygen as in cyanide toxicity [3]. Here, we describe a case of global myocardial hypoxia secondary to a suicide attempt by strangulation.

## Case presentation

A 21-year-old male presents to the emergency room after being found unresponsive in a suicide attempt by strangulation at a correctional facility. The patient has a history of depression treated with duloxetine as well as prior suicidal ideation. He has no known cardiovascular risk factors. At the correctional facility, the patient was unresponsive and in pulseless electrical activity (PEA). Guideline-directed advanced cardiopulmonary life support (ACLS) was initiated with six intravenous injections of 1 mg epinephrine and eventual return of spontaneous circulation.

In the emergency room, the patient was intubated with a pulse rate of 120 beats per minute and blood pressure of 144/122 mmHg. Oxygen saturation was 98% on the ventilator. Physical examination revealed an unresponsive patient with a linear, horizontal ecchymotic lesion on the neck. Cardiopulmonary examination revealed coarse breath sounds but no audible murmurs, gallops, S3/S4, or any significant jugular venous distension or lower extremity edema. Laboratory data included leukocytosis to 35.7 K/uL (reference range, 4.0-11.0 K/uL), severe acidosis with a pH of 6.71 (reference range, 7.35-7.45), anion gap of 50 (reference range, 4-16), and a high sensitivity cardiac troponin (HScT) trend-to-peak of 22 ng/L to 128 ng/L, to finally peak of 180 ng/L (reference range, <19 ng/L). Serum sodium and potassium were within normal limits. Chest x-ray revealed an 8 mm right apical pneumothorax with normal tracheal alignment and no consolidations present. A computed tomography scan of the head revealed severe anoxic brain injury with diffuse cerebral edema.

**Figure 1:**
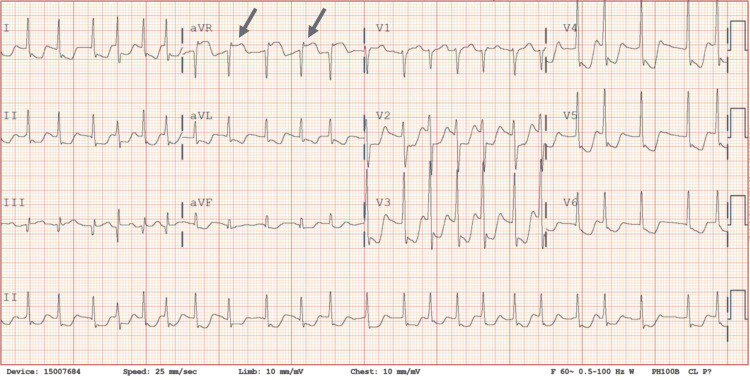
Initial electrocardiogram showing atrial fibrillation with diffuse ST-depression in Leads I and II, and precordial leads V2-V6. Further, note the significant ST-elevation in aVR (arrows). aVR: augmented vector right; aVL: augmented vector left; aVF: augmented vector foot

A 12-lead electrocardiogram (ECG) revealed atrial fibrillation with diffuse horizontal and/or downsloping ST depression (STD) of approximately 5 mm in leads I, augmented vector left (aVL), and II and ST depression of 3-6 mm in leads V2-V6 with a 3 mm ST-elevation in lead augmented vector right (aVR) (Figure 1). 

**Figure 2:**
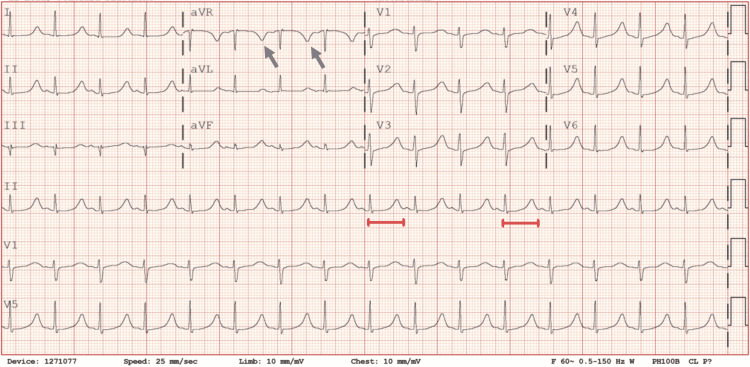
Subsequent electrocardiogram revealed sinus rhythm with normal ST segments. Note the new prolonged QT intervals (red lines) and inverted T-waves in aVR (arrows). aVR: augmented vector right; aVL: augmented vector left; aVF: augmented vector foot

Because of the anoxic brain injury and low cardiac troponin rise, cardiac catheterization was deferred, and supportive treatment was instituted. A repeat ECG revealed sinus rhythm with normalized ST segments in all leads but significant QT segment prolongation to 541 ms (QTcB reference range, 350-450 ms for adult men) (Figure 2). 

Transthoracic echocardiogram revealed an ejection fraction of 60% with no localized hypokinesis/akinesis. On the fourth hospital day, the patient was pronounced brain dead from the extensive anoxic brain injury and unable to recover.

## Discussion

The initial ECG pattern of diffuse downsloping STD with ST segment elevation (STE) in aVR that is greater than that in lead V1, has been associated with a 75% predictive accuracy for a left main coronary artery (LMCA) stenosis or multivessel disease [1]. A critical proximal left anterior descending artery (LAD) stenosis has also been associated with a similar ECG pattern [1]. However, a recent retrospective study by Harhash et al. revealed that only 10% of patients with STE in aVR and diffuse STD had acute coronary occlusion [8]. Coronary artery disease (CAD) risk is absent in this patient, making obstructive coronary disease an unlikely cause of the ECG findings. Furthermore, the absence of regional wall motion abnormalities on echocardiogram makes myocardial injury unlikely in this 21-year-old male. A spontaneous coronary artery dissection (SCAD) of the LMCA with possible extension into the proximal LAD could also explain this ECG finding [1,9]; however, echocardiogram and level of biomarker rise mitigate against this. SCAD is rare in males and more often associated with STE than STD [1]. Another potential cause of transient global ischemia on ECG includes an anomalous left main coronary artery whose diagnosis is typically obtained by coronary angiogram or computed tomography angiography, neither of which was performed on this patient [10]. Also on the differential would be a posterior lateral infarct which demonstrates STD in the anterior/lateral leads with reciprocal changes in inferior leads [11]; however, once again, the lack of injury on echocardiogram and the extent of cardiac markers rise argues against this sort of myocardial injury/infarction. The patient’s history of suicidal attempt by strangulation and resolution of the ECG findings with mechanical ventilation makes profound systemic hypoxia the most likely cause of the ECG presentation.

The common non-hypoxic causes of STD and T-wave inversion (TWI) include left ventricular hypertrophy (LVH), left and right bundle branch blocks (LBBB, RBBB), hypokalemia, and digoxin effect [3-7]. Although the STD in LVH and LBBB is downsloping and greater than 1 mm, it is usually limited to the left lateral leads (I, aVL, V5, V6) and the TWI is asymmetric and discordant with the net QRS deflection [3,6]. However, ischemic TWI is usually symmetric and concordant with the QRS complex. Furthermore, this patient has no echocardiographic findings of LVH. Although prolongation of the QTc was present on the subsequent ECG taken in this case, the absence of T wave flattening and U waves on this patient’s ECG with normal serum electrolytes make hypokalemia an unlikely cause [6]. Lastly, the STD seen due to digoxin effect has a sagging pattern unlike the downsloping morphology seen with ischemia [6]. Interestingly, this patient had a normal ejection fraction despite the profound hypoxia and acidemia he suffered. This is an unusual finding because some degree of myocardial dysfunction is common and expected following a cardiac arrest. Perhaps his young age and absence of significant CAD risk factors were mitigating.

## Conclusions

We present a case of diffuse ST segment changes mimicking myocardial ischemia that originate from global hypoxia in the setting of strangulation. When investigating the ECG after a cardiac arrest, clinicians must be aware of the potential for primary coronary events to be the culprit. However, in patients with a low pre-test probability for CAD, we should also maintain a broad differential and recall other hypoxic and non-hypoxic mimickers of myocardial infarction on ECG.
